# Work-family conflict and time use: psychometric assessment of an instrument in ELSA-Brazil

**DOI:** 10.1590/S1518-8787.2016050005892

**Published:** 2016-06-28

**Authors:** Karina Araujo Pinto, Greice Maria de Souza Menezes, Rosane Härter Griep, Keury Thaisana Rodrigues dos Santos Lima, Maria da Conceição Almeida, Estela M L Aquino

**Affiliations:** I Escola de Enfermagem. Universidade Federal da Bahia. Salvador, BA, Brasil; II MUSA Programa Integrado em Gênero e Saúde. Instituto de Saúde Coletiva. Universidade Federal da Bahia. Salvador, BA, Brasil; III Laboratório de Educação em Ambiente e Saúde. Instituto Oswaldo Cruz. Fundação Oswaldo Cruz. Rio de Janeiro, RJ, Brasil; IV Maternidade Climério de Oliveira. Universidade Federal da Bahia. Salvador, BA, Brasil

**Keywords:** Psychometrics, methods, Conflict (Psychology), Family, Work, Reproducibility of Results

## Abstract

In this study, we evaluated the psychometric properties of the items to measure the work-family conflict and the time use for personal care and leisure, included in the baseline questionnaire of the Longitudinal Study of Adult Health (ELSA-Brazil). We evaluated temporal stability (7-14 days) using kappa statistic and the validity of the construct by the correlation of Kendall’s tau with other variables. Test-retest stability was discreet to moderate and the correlations were compatible with the underlying theory. Future studies in the context of ELSA-Brazil and in other populations will complement the assessment of its relevance.

## RESUMO

Neste estudo, avaliamos as propriedades psicométricas dos itens para mensurar o conflito trabalho-família e o uso do tempo para cuidado pessoal e lazer, incluídos no questionário da linha de base do Estudo Longitudinal de Saúde do Adulto (ELSA-Brasil). Foram avaliadas a estabilidade temporal (7-14 dias) utilizando estatística kappa e a validade do construto pela correlação tau de Kendall com outras variáveis. A estabilidade teste-reteste foi discreta a moderada e as correlações, compatíveis com a teoria subjacente. Estudos futuros no contexto do ELSA-Brasil e em outras populações complementarão a avaliação da sua pertinência.

## INTRODUCTION

The blurred boundaries between work and personal life, in addition to the shortage of time, have been regarded as central issues in sociological studies^5^. In this context arises the concept of work-family conflict (WFC), characterized by the conflict between roles in which the demands of work and family, in some aspect, are irreconcilable^1,3,4^. It has a two-dimensional character (work affecting family life or vice versa) and emerges, when efforts to meet work demands interfere with ability to respond to the demands of family, or vice versa^3^. Time use was incorporated in more recent studies considering its importance in everyday choices and lifestyles, as well as being related to the roles performed in contemporary society^5^.

Efforts to perform daily activities in different social roles are presented differently for women and men and can influence their behaviors for health maintenance. Gender asymmetries regarding the time use for personal care and leisure can produce effects on the physical and mental health still little known^5^. In international literature, there is evidence of association between WFC and increased consumption of alcohol, unhealthy eating habits, decreased physical activity, obesity, exhaustion, anxiety, depression, and sleep disorders, among other health outcomes^3,4^.

The international literature on WFC is vast and there is no consensus on the best instrument to its measurement. Some studies employ isolated items, while others propose scales that do not always include the different aspects of the conflict^3,4^. We highlight the absence of instruments adapted to Brazilian Portuguese, as well as items that measure the interference of the demands of work and family about time use for personal care and leisure. Thus, three items were incorporated into the measurement of WFC and an item about the perception of time use for personal care and leisure in a multidimensional questionnaire applied to the baseline of the Longitudinal Study of Adult Health (ELSA-Brazil). This article reports the test-retest reliability and construct validity of the items among the participants of the study.

## METHODS

ELSA-Brazil is a cohort study carried out in six Brazilian state capital cities, whose population of study consists of 15,105 public servers of both sexes, aged between 35 and 74 years in the baseline^2^. For this study, we considered the 12,097 active servers.

The WFC was measured by means of three items adapted from an international instrument^4^. The items assessed to what extent the work affects family, concerning time: “Work demands prevent people from spending the desired amount of time with the family” – and regarding wear: “Work demands hinder compliance with domestic responsibilities such as taking care of the house and children”. They also assessed the extent to which family affects work: “Family demands interfere in the professional responsibilities, as an example, arrive promptly, fulfill tasks, not to miss appointments, travel for work and attend meetings outside the regular hours”. A fourth item measure the interference of work and family in personal care and leisure: “Professional and family demands prevent people from using the desired time for their own care and leisure” – developed by the authors of this article, based on previous studies that showed relevant gender differences regarding the time use^5^. The participants declared their degree of agreement with the five alternatives provided for each items, ranging from “never or almost never” to “very often”, with scores from 0 to 4.

Intraobserver reliability study was conducted using test-retest study, with range of seven to 14 days, in a convenience sample (n = 220). In the analysis, the kappa statistic was applied with quadratic weighting (kw^2^). We use the following classification for the interpretation of the values: < 0.10 – virtually absent reliability; 0.10 to 0.40 – weak; 0.41 to 0.60 – discreet; 0.61 to 0.80 – moderate; and 0.81 to 1.0 – substantial^6^.

The validity of the construct was evaluated separately for men and women by means of correlation between the items and construct-related variables identified in the literature on the subject: professional work (working hours, demand, control over work and social support at work) and family (marriage, children, taking care of someone sick or disabled at home, and having a salaried housemaid). We used Kendall tau rank correlation coefficient, with 95% confidence intervals. For each item, we verified the direction of the correlation between Kendall’s tau and the variables related to work and family, to compare them with the correlations foreseen on the literature^1,3,4^.

ELSA-Brazil was approved by the Research Ethics Committees of each of the six Research Centers and by the National Committee of Research Ethics (CEP/CONEP system – Process 13065).

## RESULTS

We observed similar sociodemographic characteristics between the baseline population of ELSA-Brazil and the subsample of test-retest reliability study.

Three items showed moderate temporal stability: “Work demands prevent people from spending the desired amount of time with the family” (kappa = 0.63; 95%CI 0.52–0.71); “Work demands hinder compliance with domestic responsibilities such as taking care of the house and children” (kappa = 0.56; 95%CI 0.45–0.67); “Professional and family demands prevent people from using the desired time for their own care and leisure” (kappa = 0.70; 95%CI 0.63–0.77). One item had discrete stability: “Family demands interfere in the professional responsibilities, as an example, arrive promptly, fulfill tasks, not to miss appointments, travel for work and attend meetings outside the regular hours” (kappa = 0.46; 95%CI 0.32–0.58).

Analyzing construct validity, we observed that, of the variables correlated to the family domain, being married or living together, having children, and taking care of dependent who requires special care were positively related to all items tested, among women. Among men, being married or living together and having children showed positive correlation with most of the items evaluated. The taking care of dependent who requires special care variable showed no significant correlation with any of the items. For men and women, we observed negative correlation between not having a salaried housemaid and all items of conflict between work and family. All work-related variables showed statistically significant positive correlation with the four items tested, both for women and men (Table).

**Table:** Correlation between the items for measurement of work-family conflict and time use for personal care and leisure, and theoretically relevant variables, according to sex, in the baseline of ELSA-Brazil. ELSA-Brazil, 2011.

Theoretically relevant variable	1. WFC^a^ based on time		2. WFC^a^ based on wear		3. WFC^b^ based on wear		4. WFD^c^ x time use for personal care and leisure	
			
	M	F	M	F	M	F	M	F
Being married or living in a stable union	0.04^d^	0.07^d^	0.01	0.07^d^	0.03^d^	0.05^d^	0.03^d^	0.10^d^
Having children	0.02	0.05^d^	-0.02	0.07^d^	0.03^d^	0.08^d^	-0.01	0.08^d^
Taking care of someone sick or disabled	0.01	0.04^d^	0.00	0.03^d^	0.01	0.07^d^	0.02	0.07^d^
Not having a salaried housemaid	-0.13^d^	-0.13^d^	-0.12^d^	-0.11^d^	-0.12^d^	-0.12^d^	-0.16^d^	-0.15^d^
Weekly working hours > 40 hours	0.32^d^	0.30^d^	0.27^d^	0.24^d^	0.10^d^	0.04^d^	0.28^d^	0.21^d^
High demand of work	0.29^d^	0.29^d^	0.26^d^	0.26^d^	0.15^d^	0.09^d^	0.26^d^	0.21^d^
Low control over work	0.14^d^	0.14^d^	0.13^d^	0.10^d^	0.09^d^	0.05^d^	0.15^d^	0.09^d^
Low social support at work	0.14^d^	0.11^d^	0.15^d^	0.16^d^	0.11^d^	0.10^d^	0.18^d^	0.16^d^

ELSA: Longitudinal Study of Adult Health; WFC: work-family conflict; WFD: Work-family demands; M: male; F: female

^a^ Conflict of work towards family.

^b^ Conflict of family towards work.

^c^ Work and family demands.

^d^ p < 0.05.

## DISCUSSION

The temporal stability of the items indicated satisfactory performance, and the small amplitudes of the confidence intervals ensured good accuracy of the estimated values. The consistency of the answers to the test and the retest ranged between discreet and moderate^6^, a result considered acceptable for reliability studies using kappa statistic. In addition, the items evaluated were correlated to variables related to work, in accordance with the literature^1,3,4^.

The results of the positive correlations among all items of WFC and being married or having children, in addition to taking care of someone sick or disabled, among women, are consistent with the literature^1^. The negative correlation found between “not having a salaried housemaid” (which presupposes more time dedicated to housekeeping work) and all items tested here stood out, for men and women, which differs from previous results that went on the opposite direction. We believe that cultural differences related to gender inequalities between Brazil and other countries may partly explain these findings^1,5^. However, such differences need to be explored in subsequent studies, considering the characteristics of the family, education, income, among other aspects.

As far as it was possible to investigate, ELSA-Brazil was the first study to include questions about WFC in Brazil. This inclusion in a study involving an extensive sample of Brazilian workers allows us to explore unprecedentedly the relationship of WFC with different health outcomes. We also highlight that the item on the influence of work and family demand on the perception of time to personal care allows the expansion of the ability to capture the perception of conflict in synchronization of social times inherent in modern life and its influence on health^5^. Furthermore, the items incorporate the most important aspects to the study of WFC: directionality and the differentiation between the conflict of work on family based in time and wear^1,3,4^.

The results refer to a particular population and therefore are limited concerning generalizations. However, they contribute to scientific knowledge for preventing results of a context outside the axis of developed countries, from where the majority of the studies come. It is possible that the few items used represent partially the work-family conflict model and therefore require further development. However, even the most extensive available instruments did not have the capacity to capture all facets involved in the construct^3^. This explains the diversity of existing proposals and the lack of consensus on the best way to do it. Due to the importance of the subject and the scarcity of studies on Brazil, we highlight the need to develop a line of investigation that can measure the construct work-family conflict in its complexity in the Brazilian context and contribute to this international debate.
